# Uncovering a Nuisance Influence of a Phenological Trait of Plants Using a Nonlinear Structural Equation: Application to Days to Heading and Culm Length in Asian Cultivated Rice (*Oryza Sativa* L.)

**DOI:** 10.1371/journal.pone.0148609

**Published:** 2016-02-09

**Authors:** Akio Onogi, Osamu Ideta, Takuma Yoshioka, Kaworu Ebana, Masanori Yamasaki, Hiroyoshi Iwata

**Affiliations:** 1 Department of Agricultural and Environmental Biology, Graduate School of Agricultural and Life Sciences, The University of Tokyo, Tokyo, Japan; 2 Western Region Agricultural Research Center, National Agriculture and Food Research Organization, Fukuyama, Hiroshima, Japan; 3 Food Resources Education and Research Center, Graduate School of Agricultural Science, Kobe University, Kasai, Hyogo, Japan; 4 Genetic Resources Center, National Institute of Agrobiological Sciences, Tsukuba, Ibaraki, Japan; Wageningen University, NETHERLANDS

## Abstract

Phenological traits of plants, such as flowering time, are linked to growth phase transition. Thus, phenological traits often influence other traits through the modification of the duration of growth period. This influence is a nuisance in plant breeding because it hampers genetic evaluation of the influenced traits. Genetic effects on the influenced traits have two components, one that directly affects the traits and one that indirectly affects the traits via the phenological trait. These cannot be distinguished by phenotypic evaluation and ordinary linear regression models. Consequently, if a phenological trait is modified by introgression or editing of the responsible genes, the phenotypes of the influenced traits can change unexpectedly. To uncover the influence of the phenological trait and evaluate the direct genetic effects on the influenced traits, we developed a nonlinear structural equation (NSE) incorporating a nonlinear influence of the phenological trait. We applied the NSE to real data for cultivated rice (*Oryza sativa* L.): days to heading (DH) as a phenological trait and culm length (CL) as the influenced trait. This showed that CL of the cultivars that showed extremely early heading was shortened by the strong influence of DH. In a simulation study, it was shown that the NSE was able to infer the nonlinear influence and direct genetic effects with reasonable accuracy. However, the NSE failed to infer the linear influence in this study. When no influence was simulated, an ordinary bi-trait linear model (OLM) tended to infer the genetic effects more accurately. In such cases, however, by comparing the NSE and OLM using an information criterion, we could assess whether the nonlinear assumption of the NSE was appropriate for the data analyzed. This study demonstrates the usefulness of the NSE in revealing the phenotypic influence of phenological traits.

## Introduction

Phenological traits of plants, such as flowering time and days to maturity, are tightly linked to growth phase transition. It is well established that phenological traits have causal influences on other traits through the modification of the duration of growth period. These causal influences result in phenotypic correlations between the phenological traits and the influenced traits. For example, significant phenotypic correlations between the days to flowering and other agronomic traits have been reported for rice [1–4], wheat [5, 6], sorghum [7, 8], meadow fescue [9], and cotton [10]. The days to maturity in soybean often correlate with other traits including seed yield [11, 12]. These phenotypic correlations suggest a causal influence of the phenological traits. However, it is usually unclear to what extent such correlations are explained by the influence as the correlation also can be generated by genetic factors (pleiotropic effects of genes or linkage disequilibrium (LD) between monotropic genes) and environmental factors.

The influence of the phenological trait is a nuisance in plant breeding because it hampers genetic dissection and evaluation of the influenced traits [4]. The genetic effects (major gene and polygenic effects) on the influenced traits contain two components, one that directly affects the traits and one that indirectly affects the traits via the phenological trait. These cannot be distinguished by phenotypic evaluation or by ordinary statistical methods. Consequently, with respect to genetic dissection, the influence of the phenological trait causes the quantitative trait loci (QTLs) responsible for the phenological traits to be identified as the candidate QTLs of the influenced traits [2–4]. However, it is unclear whether the identified QTLs control the influenced traits directly or indirectly (i.e., via the phenological trait). With respect to the genetic evaluation, because the direct genetic effects on the influenced traits are contaminated by the effects on the phenological trait, the phenotypes of the influenced traits can change unexpectedly when the phenological trait is modified by introgression or editing of the responsible genes. Thus, uncovering the influence of the phenological trait and purifying the genetic effects concealed by the phenological trait are important in plant breeding.

The causal relationships between traits can be evaluated statistically using multiple trait regression models encompassed in structural equation models (SEMs). These are comprehensive regression models for complex and multivariate data. In general, SEMs consist of two components: structural equations that describe the relationships between the latent variables, and measurement equations that combine the latent variables with the observed variables [13]. A structural equation is characterized by the dependent variables that are used as explanatory variables for other dependent variables. Such an equation can be used to infer the causal influence of a trait on other traits. In the case of the phenological trait, the phenotypic values of the phenological trait are used both as the dependent variables and the explanatory variables of the influenced traits. An example of a structural equation in which two traits are involved is,

$$\left[\begin{array}{c} y_{i,P}\\ y_{i,A} \end{array}\right]=\left[\begin{array}{cc} 0 & 0\\ \lambda & 0 \end{array}\right]\left[\begin{array}{c} y_{i,P}\\ y_{i,A} \end{array}\right]+\left[\begin{array}{c} u_{i,P}\\ u_{i,A} \end{array}\right]+\left[\begin{array}{c} e_{i,P}\\ e_{i,A} \end{array}\right]$$ (1)

where *y*_*i*, *P*_ and *y*_*i*, *A*_ are the phenotypic values of line *i* of the phenological and influenced traits *P* and *A*, respectively, *λ* represents the magnitude of the influence of the phenological trait, *u*_*i*, *P*_ and *u*_*i*, *A*_ are the “direct” genetic effects on each trait, and *e*_*i*, *P*_ and *e*_*i*, *A*_ are the residuals. If the influence *λ* is not modeled explicitly as in the ordinary regression models, the genetic effect on trait *A* is contaminated by the effect on the phenological trait *P*. That is, we obtain *λu*_*i*, *P*_ + *u*_*i*, *A*_ as the genetic effect on trait *A*. To date, several studies in plant and animal breeding have used structural equations to infer the causal influences between traits; for example, between yield component traits and the yield in wheat [14] and between somatic cell score/mastitis and milk yield in dairy cattle [15–17]. However, structural equations have not yet been applied to phenological traits.

A major drawback of a structural equation, such as illustrated in Eq (1), is that it cannot consider the nonlinear relationships between the traits. However, phenological traits can have nonlinear influences. Various environmental factors, including the climatic conditions and the amount of available nutrition, would change in addition to the timing of growth phase transitions as the phenological traits change. These factors may affect the development or emergence of other traits in a complex manner. Consequently, these factors can result in the influences of the phenological traits being nonlinear. In SEMs, parametric [18] and semi-parametric [19] approaches have been proposed to model the nonlinear relationships between the latent variables included in the structural equations. A complementary approach is to use basis expansion such as B-spline [20]. Although, in SEMs, B-spline has been used to model the nonlinearity between the dependent (endogenous) and explanatory (exogenous) variables [21, 22], it has not been used for the relationship between the dependent variables.

This study developed a nonlinear structural equation (NSE) to uncover the phenotypic influence of the phenological traits on other traits and to infer the direct genetic effects on the influenced traits. The influence was modeled using B-splines to allow nonlinear relationships between the phenological traits and the influenced traits. We applied our method to days to heading (DH) and culm length (CL) of the 110 rice cultivars (*Oryza sativa* L.) evaluated at four locations. Because DH of rice is linked to the growth phase transition from vegetation to reproduction, CL is often influenced by DH. However, the genetic evaluation of CL is usually conducted without considering this influence [23]. Thus, the major gene and polygenic effects on CL were expected to be “contaminated” by the effects on DH. The objectives of the real data analysis were to infer the influence of DH on CL and to infer the direct major genes and polygenic effects on CL. We also analyzed data sets simulating the real data. The objectives of this simulation analysis were to assess how accurately the NSE inferred the nonlinear influence of DH and the direct genetic effects on CL, and to assess the robustness of the inference of the NSE when the influence was linear or there was no influence.

## Materials and Methods

### Plant materials

We used 110 *japonica* rice cultivars including 20 landraces (S1 Table). DH and CL had been evaluated over multiple years at four locations; at the National Agriculture and Food Research Organization (NARO) Institute of Crop Science (NICS) in 2004 and 2005; at the National Institute of Agrobiological Sciences (NIAS) in 2008; at the Food Resources Education and Research Center (FRERC) of Kobe University in 2009; and at the NARO Western Region Agricultural Research Center (WARC) over seven consecutive years (2006–2012). Geographical and climatic information and seedling and transplanting dates are presented in S2 Table. Two replications per cultivar were available at FRERC and one replication was available at the other locations. We averaged the phenotypic records across the years at NICS and WARC and across the replications at FRERC. The Pearson correlation coefficients of the phenotypic values between the years and the replications were generally high; on average, 0.98 (DH) and 0.93 (CL) (S3 and S4 Tables). Two cultivars, “Norin 18” and “Nihonmasari”, were not evaluated at NIAS. The cultivar “Yukara” was not evaluated at WARC in 2010. The DH and CL records at WARC in 2006–2011 were used in our previous study assessing the accuracy of genomic prediction in rice [24]. The measurement methods of the phenotypes are described in that previous study [24].

**Fig 1:**
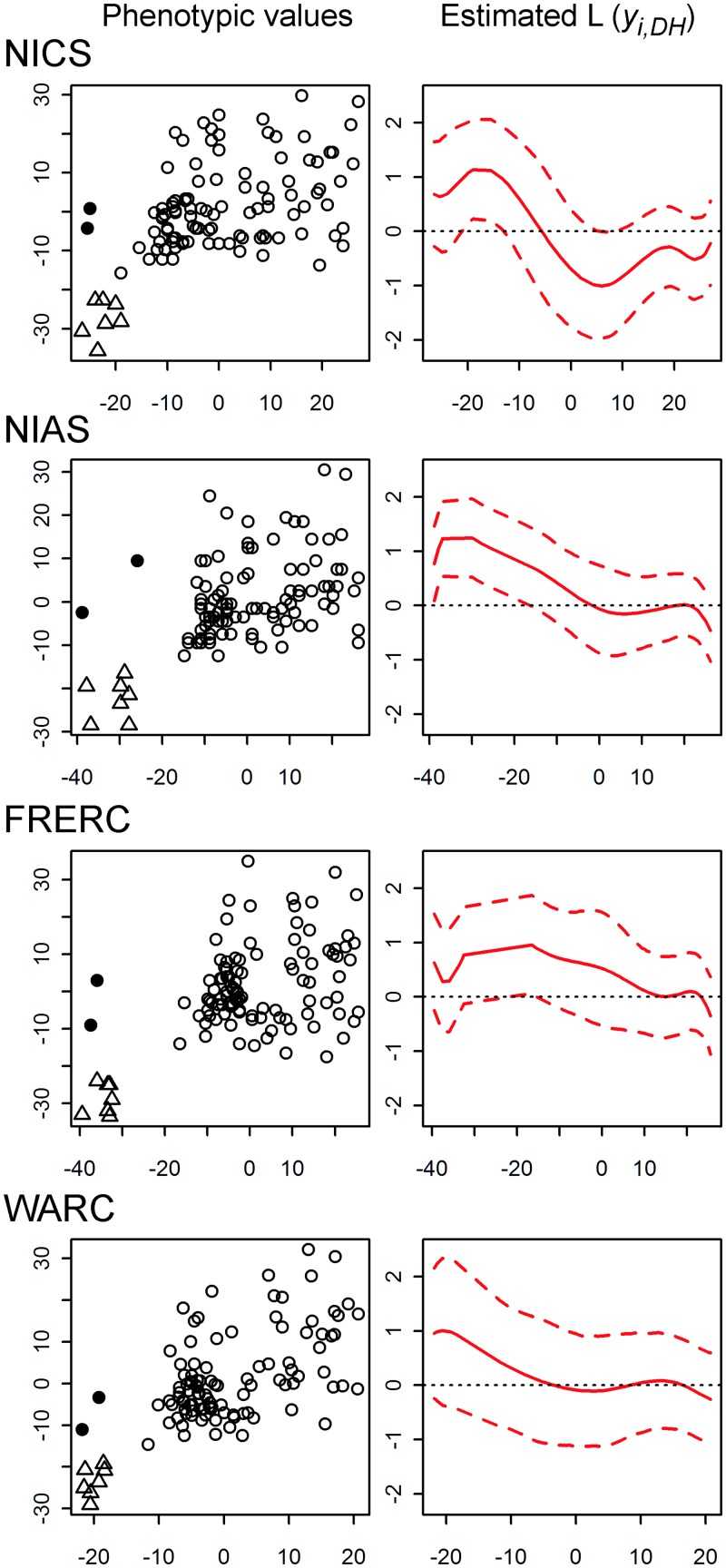
Phenotypic values of days to heading (DH) and culm length (CL) in each location (left panels) and the trajectories of the influence of DH on CL estimated by the nonlinear structural equation (right panels). The x- and y-axes of the left panels are DH (d) and CL (cm), respectively. Triangles and solid circles indicate the cultivars (“Kirara397”, “Hoshinoyume”, “Yukihikari”, “Hayamasari”, “Hatsushizuku”, “Yukara” and “Eiko”) and the landraces (“Akage” and “Bozu”) adapting to Hokkaido. The x- and y-axes of the right panels are DH (d) and the influence of DH on CL (cm/d), respectively. The red solid and dashed lines indicate the posterior means of the influence of DH and the 95% quantile lines of the posterior distributions, respectively. The dotted lines indicate zero (no influence).

Among the 110 cultivars, seven cultivars (“Kirara397”, “Hoshinoyume”, “Yukihikari”, “Hayamasari”, “Hatsushizuku”, “Yukara” and “Eiko”) were improved in Hokkaido, which is the northernmost region in Japan, and two cultivars (“Akage” and “Bozu”) were the landraces considered to be unique to this region (S1 Table). These cultivars are considered to have little photosensitivity to adapt to the cold climate of Hokkaido. In fact, they showed extremely short DH in all the locations used in this study (Fig 1) because the experimental fields were located at lower latitudes than Hokkaido (S1 Table) and the climates were warmer. We refer to these nine cultivars as the early-heading cultivars. The early-heading cultivars showed shorter CL than the other cultivars, suggesting DH had a causal influence on CL in these cultivars. We expected that the NSE would infer this known influence correctly. However, the influence of DH was less clear in the other cultivars, and the aim of the proposed method was to infer the unknown relationship between the traits in these cultivars.

### Marker information and major gene genotypes

Genotypes of 3,102 genome-wide bi-allelic markers were available for the cultivars [24]. The details of DNA extraction, genotyping, and the marker information were provided in the previous study report [24].

Genotypes of five heading date genes and *SD1* had been determined (S1 Table): for *Hd6* [25], *Hd16* [26], and *Hd17* [27], genotypes of a bi-allelic polymorphism were determined for each gene. Genotypes of two bi-allelic polymorphisms were determined for *Ghd7* [28]. For *Hd1*, seven haplotypes were constructed from nine polymorphisms. Because “Aikoku” and “Taichung 65” had unique polymorphisms on the gene, the haplotypes of these cultivars were unique to them. Because it was difficult to infer the effect of a singleton mutation, we assigned to the two cultivars the haplotype *Hd1*.*2* consisting of the same polymorphisms except for the unique polymorphisms. Thus, there were five *Hd1* haplotypes in total. The genotypes of three polymorphisms in the *SD1* gene, which were originally harbored by three cultivars, “Reimei”, “Jikkoku”, and “IR8”, respectively [29], were also determined (S1 Table). The major gene effects are reported as twice the inferred allele substitution effects. The alleles of “Koshihikari” were used as the references (i.e., the estimated effect of the “Koshihikari” allele was always zero).

### Nonlinear structural equation

**Fig 2:**
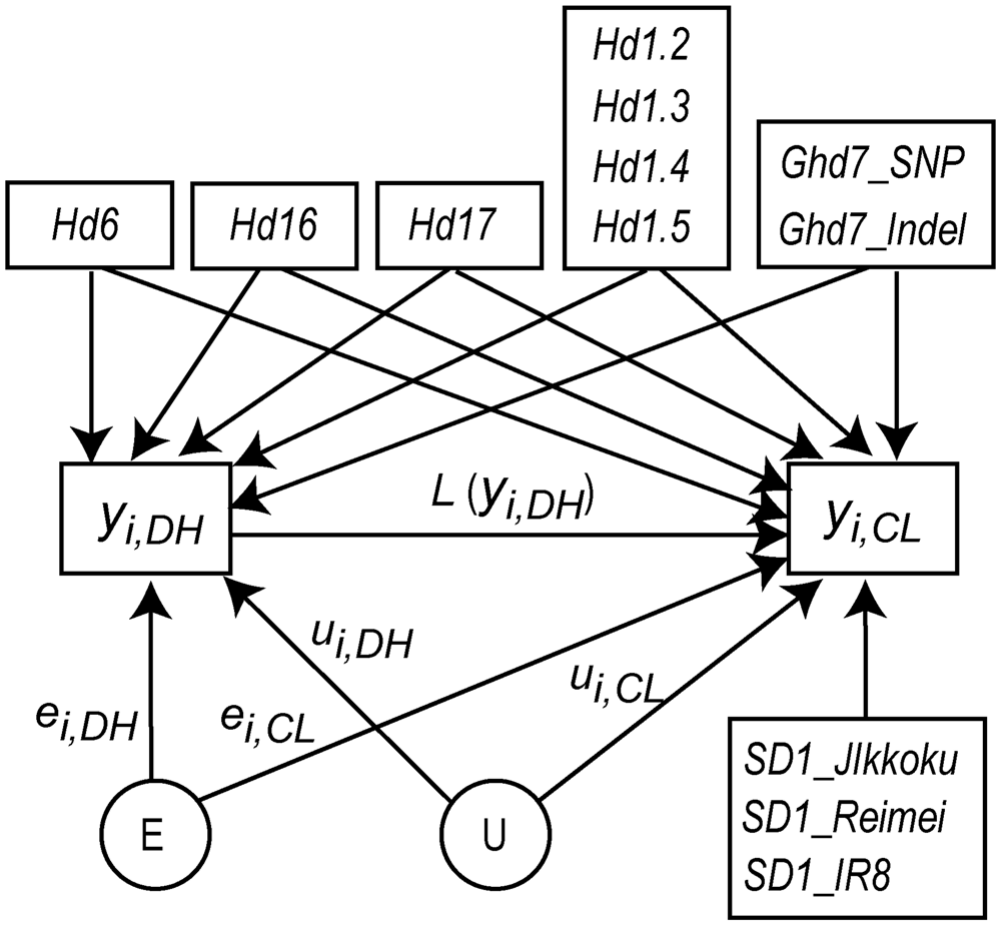
A graphical representation of the nonlinear structural equation. U and E denote the polygenic effect and residual error, respectively. The circles for U and E indicate that these nodes are unobservable, while the rectangles of the other nodes indicate that these are observable. The polymorphisms of the heading date genes were assumed to affect both days to heading (DH) and culm length (CL), whereas the polymorphisms on *SD1* gene were assumed to affect only CL. The phenotype of DH (*y*_*i*, *DH*_) was assumed to influence the phenotype of CL (*y*_*i*, *CL*_) though the function *L*, which was modeled using B-spline.

For each cultivar *i*, the NSE is written as,

$$\left[\begin{array}{c} y_{i,DH}\\ y_{i,CL} \end{array}\right]=\left[\begin{array}{cc} 0 & 0\\ L\left(y_{i,DH}\right) & 0 \end{array}\right]\left[\begin{array}{c} y_{i,DH}\\ y_{i,CL} \end{array}\right]+\left[\begin{array}{cc} x_{i,DH}^{T} & 0\\ 0 & x_{i,CL}^{T} \end{array}\right]\left[\begin{array}{c} \beta_{DH}\\ \beta_{CL} \end{array}\right]+\left[\begin{array}{c} u_{i,DH}\\ u_{i,CL} \end{array}\right]+\left[\begin{array}{c} e_{i,DH}\\ e_{i,CL} \end{array}\right]$$ (2)

where *y*_*i*, *DH*_ and *y*_*i*, *CL*_ denote the phenotypic values of DH and CL, respectively, *L* (*y*_*i*, *DH*_) represents the phenotypic influence of DH, which is assumed to be a function of *y*_*i*, *DH*_, **x**_**i**, **DH**_ and **x**_**i**, **CL**_ are vectors containing the intercepts (1) and the genotypes (0 or 1) of the major gene polymorphisms of line *i*, ***β***_***DH***_ and ***β***_***CL***_ are vectors containing the regression coefficients, *u*_*i*, *DH*_ and *u*_*i*, *CL*_ are the additive genetic effects of line *i*, and *e*_*i*, *DH*_ and *e*_*i*, *CL*_ are the residuals. The phenotypic values were adjusted for their mean values such that the averages of *y*_*i*, *DH*_ and *y*_*i*, *CL*_ were calculated as zero. **x**_**i**, **DH**_ contains the genotypes of all the heading date gene polymorphisms (*Hd1*, *Hd6*, *Hd16*, *Hd17*, and *Ghd7*), whereas **x**_**i**, **CL**_ contains the genotypes of both the heading date and *SD1* gene polymorphisms. This NSE is illustrated graphically in Fig 2. Hereafter, we refer to ***β***_***CL***_ and *u*_*i*, *CL*_ as the direct genetic effects on CL.

We applied the cubic spline to model *L (y*_*i*, *DH*_*)*;

$$L\left(y_{i,DH}\right)=\sum_{m=0}^{M}P_{m}\phi_{m}^{4}\left(y_{i,DH}\right),$$

where *P*_*m*_ is the weight of the *m*th basis function $\phi_{m}^{4}$ with order four. We set *M* = 7, indicating that eight basis functions were included in the model. As the number of basis functions increased, a more complex (meandering) trajectory could be depicted. However, because it was unlikely that the phenotypic influences of the phenological traits would become too meandering, we adopted a relatively small number (eight). A basis function of cubic spline is defined using five consecutive knots. Thus, the total number of knots was 12; the first and last four knots were arranged repeatedly at the points that were the lower and upper limits of DH, respectively; these repeated knots defined the boundaries of the B-splines [20], and the remaining four knots were arranged between these two points at equal intervals. The interval separating these four knots was determined as follows: the smallest *y*_*i*, *DH*_ value was arranged at the middle between the first point (the point where the first four knots were arranged) and the next knot. Similarly, the largest *y*_*i*, *DH*_ value was arranged at the middle between the last point (the point where the last four knots were arranged) and the preceding knot. The positions of the knots and the knot interval were determined for each location. The definition of $\phi_{m}^{4}$ followed that by Hastie et al. [20].

We also fitted an ordinary bi-trait linear model (OLM) to the data. This did not contain the term representing the phenotypic influence of DH, i.e., the first term on the right hand side of Eq (2). Note that, when OLM is fitted to the data when the influence of DH on CL actually exists, i.e., *L* (*y*_*i*, *DH*_) is not equal to zero, the direct genetic effects (***β***_***CL***_ and *u*_*i*, *CL*_) cannot be estimated accurately because the genetic effects on DH (***β***_***DH***_ and *u*_*i*, *DH*_) contaminate the effects on CL. We compared the NSE and OLM using the widely applicable information criterion (WAIC) [30]; this is recommended by Gelman et al. [31] to select models in terms of their predictive ability. Lower WAIC values indicate greater power at predicting the phenotypic values (*y*_*i*, *DH*_ and *y*_*i*, *CL*_). The NSE and OLM were each fitted to the data for each location.

### Parameter estimation

Parameters were inferred using a Markov chain Monte Carlo (MCMC) method. The prior distribution of the additive genetic effects was a multivariate normal distribution MVN(**0**, **G** ⊗ **K**), where **G** is the genetic variance-covariance matrix and **K** is the genomic relationship matrix calculated from the genome-wide markers using the A.mat function of the R package rrBLUP [32, 33]. The prior distribution of the residuals was MVN(**0**, **R** ⊗ **I**), where **R** is the residual variance-covariance matrix and **I** is an identity matrix with the size of the number of cultivars. The prior distributions of **G** and **R** were inverse Wishart distributions with four degrees of freedom and with scale matrices that were half of the phenotypic variance-covariance matrix, which resulted in the expectations for these distributions being the same as the scale matrices. We assigned non-informative prior distributions to the overall means and major gene effects. The prior distributions of the weights of the basis functions were as follows

$$\begin{array}{l} P_{0}\sim N\left(\begin{array}{cc} 0, & 1000\sigma_{P}^{2} \end{array}\right)\\ P_{1}\sim N\left(\begin{array}{cc} 0, & 1000\sigma_{P}^{2} \end{array}\right)\\ P_{m}\sim N\left(\begin{array}{cc} 2P_{m-1}-P_{m-2}, & \sigma_{P}^{2} \end{array}\right)\text{ for m}\geq 2 \end{array}$$

where *N* denotes the normal distribution and $\sigma_{p}^{2}$ is the variance. The prior distribution of *P*_*m*_ for *m* ≥2 is equivalent to assuming $\left(P_{m}-P_{m-1}\right)-\left(P_{m-1}-P_{m-2}\right)\sim N\left(\begin{array}{cc} 0, & \sigma_{p}^{2} \end{array}\right)$. The prior distribution of $\sigma_{p}^{2}$ is a non-informative scaled inverse-chi-square distribution. The weights were inferred using a Metropolis update procedure, which is illustrated in the Appendix. The other parameters were inferred using Gibbs sampling, treating the phenotypic values of CL adjusted for the influence of DH (i.e., y_*i*, *CL*_−L(y_*i*, *DH*_)y_*i*, *DH*_) as the dependent variable [34]. The number of iterations was 1.1 × 10^6^ with the first 0.1 × 10^6^ iterations discarded as burnin, and the sampling interval was 100. The prior distributions of the OLM were the same as those of the corresponding parameters of the NSE. The parameters of the OLM were inferred using Gibbs sampling with the MCMC conditions the same as those for the NSE. Major gene effects were judged to be non-significant if 0 lay within the 0.025 and 0.975 quantiles of the MCMC samples. The calculation was performed using a program written in C language.

### Simulation analysis

To assess the validity and accuracy of the inference by the NSE, we conducted simulation analyses based on the parameter values estimated in the real data analyses. We considered three simulation schemes. First, the posterior means of the parameters of the NSE (*P*_*m*_, ***β***_***DH***_, ***β***_***CL***_, *u*_*i*, *DH*_, *u*_*i*, *CL*_, and **R**) at each location were used to simulate data sets. By generating random residuals using **R**, 20 data sets were created for each location. We ensured that the *y*_*i*, *DH*_ values created did not exceed the lower and upper limits defined by the knots in the real data analyses. In the second and third schemes, the posterior means of the parameters of the OLM (***β***_***DH***_, ***β***_***CL***_, *u*_*i*, *DH*_, *u*_*i*, *CL*_, and **R**) at each location were used to simulate data sets. In the second scheme, the influence was assumed to be linear; *L (y*_*i*, *DH*_*)* was set to 1.2 for all *y*_*i*, *DH*_. In the third scheme, it was assumed that DH did not influence CL, i.e., *L (y*_*i*, *DH*_*)* = 0 for all *y*_*i*, *DH*_. In both the second and third schemes, 20 data sets were created for each location by generating random residuals using **R**. We analyzed these simulated data sets using the NSE and OLM. The prior distributions of these methods were determined as described earlier (Parameter estimation).

## Results

### Phenotypic correlation

Means of the observed DH and CL values and Pearson correlation coefficients between these traits are presented in S5 Table. DH was generally shorter at WARC than at the other locations because of the lower latitude and warmer climate (S2 Table). CL at WARC was also shorter than that at the other locations. The correlation coefficients were relatively high (>0.5) in all the locations, suggesting phenotypic influence of DH on CL.

### Real data analysis

We fitted the NSE to the data at each location. The inferred influence of DH on CL was positive for the early-heading cultivars at each location (Fig 1). Because the DH and CL values were adjusted so that their means were zero, the early-heading cultivars had negative DH values. Thus, the positive estimated influence of DH on CL for the early-heading cultivars indicates that early heading resulted in short CL. At NICS, however, the influence of DH became negative as DH increased. At WARC and NIAS, the influence almost vanished at DH ≈ −5 and 0, respectively. At FRERC, the influence was the most durable and vanished at DH ≈ 10. In summary, the phenotypic influence of DH on CL was prominent for the early-heading cultivars, whereas the influence became more vague for the later-heading cultivars.

**Fig 3:**
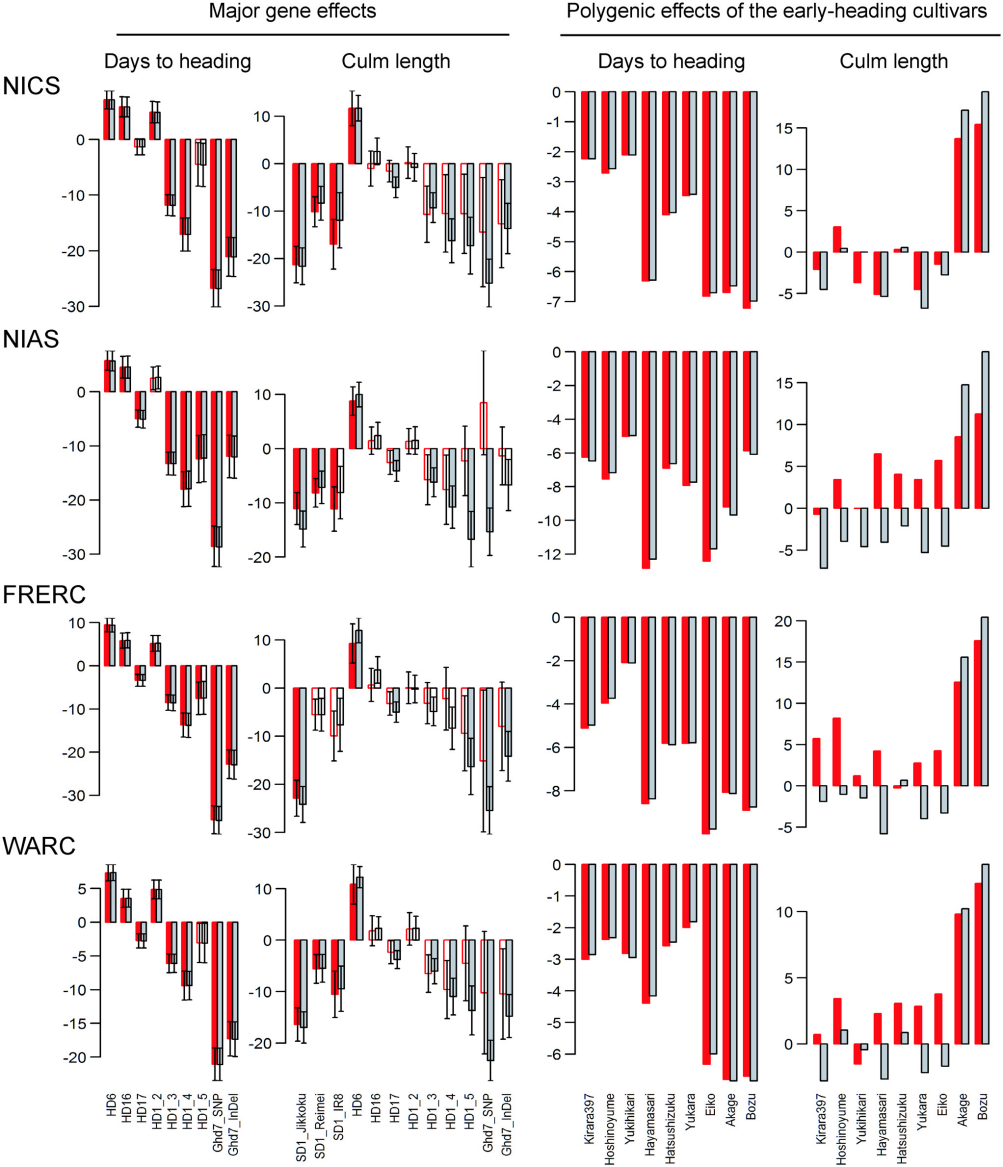
Major gene effects and polygenic effects of the early-heading cultivars estimated by the nonlinear structural equation (red) and the ordinary bi-trait linear model (gray) in the real data analyses. Major gene effects, which were not significant (P < 0.05), are represented by the outlined bars. The early-heading cultivars include the seven improved cultivars (“Kirara397”, “Hoshinoyume”, “Yukihikari”, “Hayamasari”, “Hatsushizuku”, “Yukara”, and “Eiko”) and the two landraces (“Akage” and “Bozu”).

The positive influence of DH means that the polygenic and major gene effects on CL inferred using the OLM were contaminated by the effects on DH. The contaminated effects were expected to be purified using the NSE. In Fig 3, the major gene effects estimated by the NSE and OLM are compared. The estimated gene effects on DH hardly differed between the methods. In contrast, differences were observed particularly in the effects on CL estimated for *Hd1*.*4*, *Hd1*.*5*, *Ghd7_SNP*, and *Ghd7_Indel*. In the results of the OLM, these polymorphisms have large negative effects on CL. It is reasonable that the effects were negative because the non-reference alleles at *Hd1*.*5* and *Ghd7_SNP* were unique to the early-heading cultivars (S1 Table); two early-heading cultivars had the non-reference allele at *Ghd7_Indel*, and the cultivars with the non-reference allele at *Hd1*.*4* showed earlier heading at each location (e.g., the centered DH value was −11.0 on average at NICS). Consequently, these polymorphisms had large negative effects on CL because of the phenotypic influence of DH on CL. When the influence of DH on CL was removed using the NSE, the negative effects of these polymorphisms on CL diminished (Fig 3). This suggests that the phenotypic influence of DH on CL enhanced the magnitude of these polymorphism effects on CL.

Fig 3 shows a comparison of the polygenic effects of the early-heading cultivars. Similar to the results for the major gene effects, the polygenic effects on DH hardly differed between the methods. In contrast, differences were observed in the polygenic effects on CL, particularly in the seven improved cultivars of the early-heading cultivars; the polygenic effects estimated by the NSE tended to be greater than those estimated by the OLM. This tendency was reasonable because of the following reasons: in the results of the OLM, the estimated polygenic effect on CL consisted of two components, the direct effect on CL (*u*_*i*, *CL*_ in Eq 2) and the indirect effect on CL via the influence of DH on CL (*L* (*y*_*i*, *DH*_) × *u*_*i*, *DH*_). The latter component was negative because the polygenic effect on DH (*u*_*i*, *DH*_) was negative, and the influence of DH on CL (*L* (*y*_*i*, *DH*_)) was positive. Thus, after removing this latter component by modeling the influence of DH on CL using the NSE, the polygenic effects increased. However, we also observed a contrary tendency in the two landraces of the early-heading cultivars, “Akage” and “Bozu”, which showed lesser effects on CL in the NSE analyses. We were not able to find any reason to explain this phenomenon.

We compared the NSE and OLM using mean log likelihood and WAIC (Table 1). The mean log likelihood of the NSE was larger than that of the OLM except at WARC. Similarly, the WAIC values of the NSE were lower than those of the OLM except at WARC, suggesting the superior predictive ability of NSE over OLM at all the locations except for WARC.

**Table 1:** Model comparison in real data analysis.

Location	Nonlinear structural equation		Ordinary bi-trait linear model		Delta WAIC^a^
	Mean log likelihood	WAIC	Mean log likelihood	WAIC	
NICS	−572.27	1272.93	−592.11	1310.20	−37.26
NIAS	−555.79	1235.35	−573.82	1273.30	−37.95
FRERC	−561.08	1249.90	−571.95	1270.40	−20.49
WARC	−510.19	1149.77	−507.63	1144.31	5.46

^a^The widely applicable information criterion (WAIC) value of the nonlinear structural equation subtracted from that of the ordinary two-trait linear model. Negative delta WAIC values suggest the superior predictive ability of the nonlinear structural equation.

### Simulation analyses

**Fig 4:**
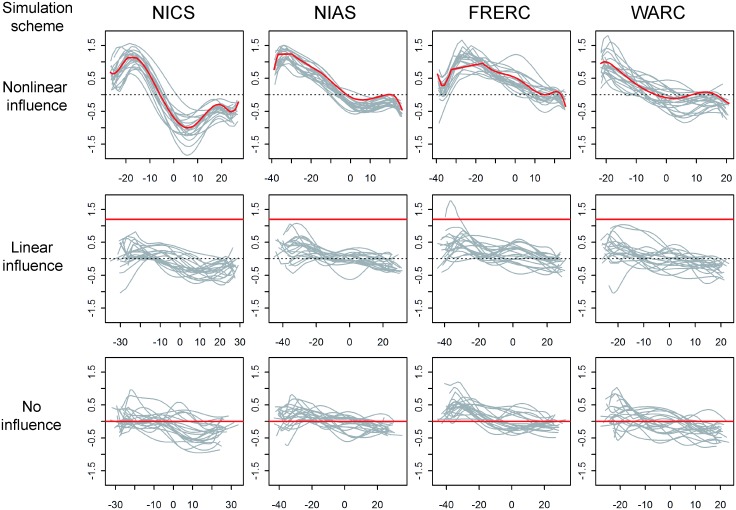
Trajectories of the influence of the days to heading (DH) on culm length (CL) estimated by the nonlinear structural equation (NSE) in the simulation analyses. We conducted three simulation schemes: “Nonlinear influence” where the nonlinear influences of DH on CL estimated in the real data analyses were used to simulate data sets; “Linear influence” where the influence was set to 1.2 for all DH values; and “No influence” where there was assumed to be no influence (i.e., zero). In each scheme, 20 data sets were simulated using the real data analysis results at each location and analyzed using the NSE. In each scheme at each location, the estimated trajectories of the influence of DH are superimposed with gray lines, and the true trajectory (value) of the influence is indicated by the red line. The dashed lines indicate zero (no influence).

We conducted simulation analyses to assess how accurately the NSE inferred the phenotypic influence of DH and the direct major gene and polygenic effects on CL. We considered three simulation schemes that differed in the phenotypic influence of DH on CL: nonlinear influences, a linear influence (*L* (*y*_*i*, *DH*_) = 1.2 for all *y*_*i*, *DH*_), and no influence (*L* (*y*_*i*, *DH*_) = 0 for all *y*_*i*, *DH*_). The inferred trajectories of the influence of DH are shown in Fig 4; the correlation coefficients between the true and estimated *L* (*y*_*i*, *DH*_) values are presented in Table 2. The NSE was able to infer the trajectory of the nonlinear influence accurately, particularly at NICS and NIAS. In contrast, the NSE failed to detect the linear influence at all the locations (Fig 4). When no influence was simulated, the inferred trajectories did not greatly deviate from zero at any DH value (Fig 4).

**Table 2:** Summary of the simulation analysis.

Scheme^a^	Location	Mean (SD) correlation coefficients between the estimated and true values					Mean (SD) delta WAIC^e^
		*L* (*y*_*i*, *DH*_)^b^	*u*_*i*, *CL*_^c^		*β*_*CL*_^d^		
		NSE	NSE	OLM	NSE	OLM	
Nonlinear	NICS	0.97 (0.02)	0.87 (0.02)	0.79 (0.03)	0.95 (0.03)	0.89 (0.03)	−76.76 (30.03)
	NIAS	0.98 (0.02)	0.85 (0.05)	0.74 (0.06)	0.88 (0.11)	0.31 (0.07)	−61.64 (27.10)
	FRERC	0.90 (0.10)	0.93 (0.02)	0.87 (0.03)	0.94 (0.05)	0.90 (0.02)	−43.95 (18.74)
	WARC	0.82 (0.16)	0.89 (0.03)	0.87 (0.03)	0.94 (0.03)	0.90 (0.03)	−14.90 (12.05)
Linear	NICS		0.65 (0.07)	0.72 (0.05)	0.87 (0.03)	0.89 (0.02)	−0.08 (18.80)
	NIAS		0.32 (0.11)	0.40 (0.10)	0.83 (0.03)	0.84 (0.02)	6.54 (12.22)
	FRERC		0.64 (0.07)	0.64 (0.06)	0.86 (0.04)	0.88 (0.02)	−2.17 (8.69)
	WARC		0.64 (0.10)	0.67 (0.08)	0.92 (0.03)	0.93 (0.02)	4.76 (11.41)
No influence	NICS		0.86 (0.03)	0.88 (0.02)	0.94 (0.04)	0.97 (0.02)	5.03 (11.14)
	NIAS		0.81 (0.03)	0.85 (0.03)	0.93 (0.05)	0.97 (0.02)	5.58 (8.18)
	FRERC		0.88 (0.02)	0.90 (0.02)	0.90 (0.11)	0.98 (0.01)	0.43 (11.58)
	WARC		0.87 (0.03)	0.90 (0.02)	0.96 (0.03)	0.99 (0.01)	3.10 (8.90)

^a^In each scheme, 20 data sets were simulated using the results of real data analysis at each location and analyzed using the nonlinear structural equation (NSE) and the ordinary bi-trait linear model (OLM); Nonlinear, assumed a nonlinear influence of days to heading (DH) on culm length (CL); Linear, *L* (*y*_*i*, *DH*_) was set to 1.2 for all *y*_*i*, *DH*_; No influence, *L* (*y*_*i*, *DH*_) was set to 0 for all *y*_*i*, *DH*_.

^b^The phenotypic influence of DH on CL of each cultivar.

^c^Polygenic effects of each cultivar on CL.

^d^Major gene effects on CL.

^e^The widely applicable information criterion (WAIC) value of NSE subtracted from that of OLM.

As the NSE considers the influence of DH on CL in its model, it was expected to infer the direct major gene and polygenic effects on CL more accurately than the OLM. Indeed, when nonlinear influences were simulated, this was the case (Table 2). In contrast, when the linear influence or no influence was simulated, the accuracy of the NSE tended to be inferior to that of the OLM. The differences in accuracy between these methods were clearly reflected by the differences in WAIC; the WAIC values for the NSE were clearly lower than those for the OLM when the nonlinear influence was simulated, whereas the values for the NSE were similar to or greater than the values for the OLM when simulating the linear influence or no influence. These results suggest the WAIC was useful for assessing whether the NSE was an appropriate model for the data sets analyzed.

## Discussion

We proposed a NSE, based on B-spline, to infer the nonlinear phenotypic influence of a phenological trait on other traits. We applied the NSE to DH and CL of cultivated rice and clarified the trajectories of the influence of DH on CL. The NSE enabled the estimation of direct major gene and polygenic effects on CL concealed by the influence from DH. The simulation study showed that the NSE was able to infer the nonlinear phenotypic influence of DH with reasonable accuracy although the sample size in this study was relatively small (110, in total). However, the NSE failed to infer the linear influence. When no phenotypic influence was simulated, the ordinary multiple-trait linear model, OLM, tended to infer genetic effects more accurately. However, the simulation study also shows the possibility of assessing whether the nonlinear assumption of NSE was appropriate for the data analyzed by comparing the WAIC values between the NSE and OLM.

The reason why NSE failed to detect the linear influence is probably related to an issue of parameter identifiability. In Eq (2), if *L* (*y*_*i*, *DH*_) is replaced by a scalar value λ, the parameters λ and the residual covariance are not identifiable because only a single information source, the phenotypic covariance between the traits, is available to determine these two parameters. To ensure identifiability between λ and the residual covariance, a constraint is required. A typical constraint is fixing the residual covariance to zero, which produces a sufficient rule of parameter identifiability called the “recursive rule” by Bollen [13]. In the NSE, however, the weight parameter *P*_*m*_ and residual covariance were identifiable without constraints on the residual covariance. In the real data analysis, the correlation of the MCMC samples between the weights and the residual covariance was weak; the mean (SD) Pearson correlation coefficients were −0.19 (0.12), −0.23 (0.09), −0.17 (0.09), and −0.20 (0.12), at NICS, NIAS, FRERC, and WARC, respectively. When the parameters are not identifiable, we usually observe a high negative correlation between the MCMC samples; for example, it can reach −0.996 [35]. Although prior information can temper identifiability in a Bayesian analysis, it was confirmed that the weight parameters and the residual covariance were identifiable when a non-informative Wishart distribution was assigned to the residual covariance; the correlation coefficients were −0.15 (0.10), −0.10 (0.06), −0.09 (0.03), and −0.21 (0.12), at NICS, NIAS, FRERC, and WARC, respectively. We speculate that the mechanism for this identifiability of NSE is as follows: when the phenotypic influence of DH is nonlinear, the weight parameter values differ from each other. These weights parameters generate *L* (*y*_*i*, *DH*_) values unique to cultivars, which would yield the phenotypic covariance between the traits unique to the cultivars. This would increase the information sources to determine the weights and residual covariance, and would make these parameters identifiable. In contrast, when the influence was linear, i.e., *L* (*y*_*i*, *DH*_) was a single value for all *y*_*i*, *DH*_, the phenotypic covariance was a constant among the cultivars. Consequently, the parameters became unidentifiable, and thus the NSE probably failed to detect the linear trend. When we simulated data sets with linear influences and no residual covariance, the NSE successfully detected the linear trajectory by imposing the constraint of being zero on the residual covariance (data not shown). The validity of assuming no residual covariance would be case-dependent. However, we should be cautious about imposing such a strong constraint. In this study, the estimated residual covariance was positive at all the locations; the posterior means (SD) were 6.68 (4.66), 6.47 (4.24), 5.23 (3.41), and 4.83 (2.59) at NICS, NIAS, FRERC, and WARC, respectively. Thus, if the residual covariance is not taken into account, the estimate of phenotypic influence will be overestimated.

In the real data analysis, the WAIC supported the superiority of the OLM only at WARC (Table 1). If we follow the simulation results, two explanations can be considered: either there was a linear influence between DH and CL or no influence existed. However, we have concluded that the following explanation is more likely. Because the phenotypic influence as DH increased vanished earlier at WARC than at the other locations (Fig 1), inference of the influence of DH on CL using the NSE would not be easy at WARC and inference with the OLM would not be severely exacerbated by the influence of DH. This explanation was supported by the results of the simulation scheme that assumed a nonlinear influence. The mean correlation between the true and inferred *L* (*y*_*i*, *DH*_) values was lowest in the simulation for WARC(0.82, Table 2), suggesting the difficulty of inference using the NSE. Furthermore, the difference in WAIC values between the methods was lowest at WARC (mean delta WAIS = −14.90, Table 2), suggesting the relatively better performance of the OLM. In addition, among the 20 replications, we observed a replication with a positive delta WAIC value (9.10), although the trajectory of the influence of DH was successfully inferred (the correlation between the true and inferred *L* (*y*_*i*, *DH*_) values was 0.92).

In the real data analyses, for all the locations, the inferred trajectories of the phenotypic influence of DH on CL showed a decreasing trend as DH increased. The influence on the early-heading cultivars was positive for all the locations, suggesting that early heading shortened the CL of these cultivars. Only at NICS, the influence became negative as DH increased, suggesting that later heading was also attributable to shorter CL at this location. Although the exact reason is unclear, we speculate that the environmental conditions encountered by the later heading cultivars, such as temperature, solar radiation, and available nutrition, might not be suitable for growth and/or heading. At the other locations, the influence of DH became less prominent as DH increased, suggesting that the influence was vague for the late-heading cultivars.

By removing the phenotypic influence of DH, the direct effects of *Hd1*.*4*, *Hd1*.*5*, *Ghd7_SNP*, and *Ghd7_Indel* on CL decreased (Fig 3). However, these polymorphisms still had non-zero effects on CL. Although the effects were insignificant for all the locations, this insignificancy was probably due to the relatively larger posterior variances of the gene effects estimated by the NSE than by the OLM. Thus, the results from the NSE suggest a possible direct effect of these polymorphisms on CL. *Hd1* is involved in flowering via the regulation of *Hd3a* [36]. Although this gene’s involvement on panicle development has been reported [37], there have been no reports of its direct effect on CL. Our results suggest direct effects but further studies are required to confirm this. *Ghd7* is thought to be involved in flowering by regulating the expression of *Ehd1* and *Hd3a* [28]. However, the expression of *Ghd7* was observed in organs that seemed unrelated to flowering, such as the stems at booting stage, suggesting the possibility that *Ghd7* is also involved in the growth of the culm by processes other than via modification of the vegetative period [28]. In addition, Weng et al. [38] reported that *Ghd7* regulates the expression level of the GA2-oxidase gene *OsGA2ox6*, which controls the plant’s height, suggesting direct effects of *Ghd7* on CL. Thus, the non-zero effects of *Ghd7_SNP* and *Ghd7_Indel* on CL are consistent with these previous reports. Among the heading date genes, *Hd6* showed significant effects on CL in all the locations. *Hd6* encodes a casein kinase II α-subunit, and controls the flowering time as an enhancer of the repressor activity of *Hd1* to *Hd3a* [25, 39]. Although the involvement of this gene in development and elongation of culm has not been reported, our results suggest this to be a possibility.

Among the early-heading cultivars, the polygenic effects on CL of the seven improved cultivars (“Kirara397”, “Hoshinoyume”, “Yukihikari”, “Hayamasari”, “Hatsushizuku”, “Yukara”, and “Eiko”) tended to increase by removing the phenotypic influence of DH. Using the estimation results of the major gene and polygenic effects and the nonlinear influence of DH, the NSE can predict CL when the heading data genes are modified. For example, suppose that the non-reference alleles on *Ghd7_SNP* of the early heading cultivar, “Kirara397”, are modified to the reference (Koshihikari’s) alleles. When the modified genotype is cultivated at FRERC, DH would be prolonged for approximately 35 days because of this modification (Fig 3). As a result, the influence of DH would reduce from 0.60 to 0.48 cm/day and the expected deviation of CL from the overall mean (92.5 cm, S4 Table) would be 9.9 cm. In contrast, when the results of the OLM are used, it would be −1.2 cm. This information would be helpful for a finer-scale design of new genotypes. Although the difference (between 9.9 and −1.2 cm) does not seem large because of the relatively large posterior variance (uncertainty) of the parameters, the posterior variance probably decreases as the sample size increases.

Because genetic resources used in plant breeding usually consist of lines/cultivars adapting to diverse environments, they often show great genetic variation in the phenological traits that have a major role in local adaptation. Consequently, field experiments at a location cannot evaluate the genetic effects of the cultivars/lines that do not adapt to the location because they are masked by the influence of the phenological traits. This phenomenon was observed for the early-heading cultivars in the present study. However, it is suggested that the genetic effects concealed by the phenological traits at the target location can be evaluated by applying the NSE. Thus, the NSE could enhance the value of genetic resource in plant breeding.

## Appendix

### Metropolis algorithm to infer the weights of the basis function

The weights of the basis function, *P*_*m*_ (0 ≤ m ≤ 7), were inferred using the following Metropolis algorithm.

(1) Generate a proposal value $P_{m}^{*}$ from a normal distribution $N\left(\begin{array}{cc} P_{m}, & \sigma_{prop}^{2} \end{array}\right)$ where *P*_*m*_ is the value at the current iteration and $\sigma_{prop}^{2}$ is set to 0.04.

(2) Accept $P_{m}^{*}$ with the probability,

$$\text{min}\left[\begin{array}{cc} 1, & \frac{P\left(P_{m}^{*}\right)}{P\left(P_{m}\right)}\prod_{i}\frac{L\left(y_{i,DH},y_{i,CL}|P_{m}^{*},P_{-m},\beta_{DH},\beta_{CL},u_{i,DH},u_{i,CL},R\right)}{L\left(y_{i,DH},y_{i,CL}|P_{m},P_{-m},\beta_{DH},\beta_{CL},u_{i,DH},u_{i,CL},R\right)} \end{array}\right]$$

where *P* and *L* indicate the prior density of *P*_*m*_ and likelihood functions, respectively, and *P*_*−m*_ indicates the weights except for *P*_*m*_. The likelihood function is illustrated by Eq 37 in [34]. It is notable that a change of *P*_*m*_ does not affect the likelihood of all the cultivars because the corresponding basis function is 0 if *y*_*i*, *DH*_ is located outside the five knots that define the basis function. Thus, in the acceptance probability above, we need to evaluate the likelihood only for the cultivars that are located within the knots.

(3) Replace *P*_*m*_ with $P_{m}^{*}$ if accepted. In our experience, the acceptance rate was 0.40–0.60.

## Supporting Information

S1 Table The cultivars used and major gene polymorphism. (XLSX)

S2 Table Geographical and climatic information for the experimental fields. (PDF)

S3 Table Pearson correlation coefficients for the phenotypic values between years (NICS) or replications (FRERC). (PDF)

S4 Table Pearson correlation coefficients of the phenotypic values between years at WARC. (PDF)

S5 Table Mean values, (co) variances, and Pearson correlation coefficients of phenotypic values between DH and CL for each location. (PDF)
