# Supplementary material for: Uncovering a Nuisance Influence of a Phenological Trait of Plants Using a Nonlinear Structural Equation: Application to Days to Heading and Culm Length in Asian Cultivated Rice (Oryza Sativa L.)
Source: PLoS One. 2016 Feb 9;11(2):e0148609. doi: 10.1371/journal.pone.0148609 (PMC4747597; doi:10.1371/journal.pone.0148609)
Supplement: S2 Table — (PDF) [file pone.0148609.s002.pdf]

S2 Table Geographical and climatic information for the experimental fields

Field abbreviation	Year	Latitude / Longitude	Mean day temperature (°)/ mean day length (h)					Sowing	Trans-planting
			May	Jun.	Jul.	Aug.	Sep.		
NICS	2004–2005	N36° 01' / E140° 02'	16.5/ 14.1	21.3/ 14.6	24.9/ 14.4	25.8/ 13.6	23.1/ 12.4	2–13 May	26 May
NIAS	2008	N36° 02' / E140° 11'	16.6/ 14.1	19.7/ 14.6	25.0/ 14.4	25.3/ 13.6	22.1/ 12.4	21 Apr.	21 May
FRERC	2009	N34° 88' / E134° 86'	18.5/ 14.0	22.6/ 14.5	25.7/ 14.3	26.8/ 13.6	23.1/ 12.4	30 Apr.	8–11 Jun.
WARC	2006–2012	N34° 50' / E133° 39'	18.5/ 14.0	22.8/ 14.4	26.6/ 14.2	28.5/ 13.5	24.7/ 12.4	25 May–1 Jun.	18–24 Jun.
