# Supplementary material for: Uncovering a Nuisance Influence of a Phenological Trait of Plants Using a Nonlinear Structural Equation: Application to Days to Heading and Culm Length in Asian Cultivated Rice (Oryza Sativa L.)
Source: PLoS One. 2016 Feb 9;11(2):e0148609. doi: 10.1371/journal.pone.0148609 (PMC4747597; doi:10.1371/journal.pone.0148609)
Supplement: S3 Table — (PDF) [file pone.0148609.s003.pdf]

S3 Table Pearson correlation coefficients for the phenotypic values between years (NICS) or replications (FRERC)

Location	DH	CL
NICS	0.97	0.90
FRERC	0.99	0.95

DH, days to heading; CL, culm length
