# Supplementary material for: Uncovering a Nuisance Influence of a Phenological Trait of Plants Using a Nonlinear Structural Equation: Application to Days to Heading and Culm Length in Asian Cultivated Rice (Oryza Sativa L.)
Source: PLoS One. 2016 Feb 9;11(2):e0148609. doi: 10.1371/journal.pone.0148609 (PMC4747597; doi:10.1371/journal.pone.0148609)
Supplement: S4 Table — (PDF) [file pone.0148609.s004.pdf]

S4 Table Pearson correlation coefficients of the phenotypic values between years at WARC (upper diagonal, days to heading; lower diagonal, culm length)

	2006	2007	2008	2009	2010	2011	2012
2006		0.98	0.98	0.99	0.99	0.99	0.99
2007	0.96		0.95	0.98	0.98	0.97	0.98
2008	0.94	0.92		0.98	0.98	0.98	0.98
2009	0.91	0.93	0.85		0.98	0.99	0.99
2010	0.96	0.93	0.95	0.87		0.99	0.99
2011	0.94	0.94	0.95	0.90	0.94		0.99
2012	0.96	0.96	0.93	0.93	0.95	0.95	
