# Supplementary material for: Uncovering a Nuisance Influence of a Phenological Trait of Plants Using a Nonlinear Structural Equation: Application to Days to Heading and Culm Length in Asian Cultivated Rice (Oryza Sativa L.)
Source: PLoS One. 2016 Feb 9;11(2):e0148609. doi: 10.1371/journal.pone.0148609 (PMC4747597; doi:10.1371/journal.pone.0148609)
Supplement: S5 Table — (PDF) [file pone.0148609.s005.pdf]

S5 Table Mean values, variances (diagonal), covariances (upper off-diagonal), and Pearson correlation coefficients (lower off-diagonal) of phenotypic values in each of four locations analyzed in this study

		NICS		NIAS		FRERC		WARC	
		DH	CL	DH	CL	DH	CL	DH	CL
Mean		93.0	86.7	105.1	72.5	101.5	92.5	78.3	76.7
(Co)variance & correlation	DH	175.8	86.3	221.8	82.6	226.6	99.1	104.6	77.2
	CL	0.51	161.2	0.52	114.5	0.51	167.2	0.64	137.6
